# Cone Photoreceptors in Diabetic Patients

**DOI:** 10.3389/fmed.2022.826643

**Published:** 2022-03-17

**Authors:** Ann E. Elsner, Brittany R. Walker, Robert N. Gilbert, Vamsi Parimi, Joel A. Papay, Thomas J. Gast, Stephen A. Burns

**Affiliations:** School of Optometry, Indiana University, Bloomington, IN, United States

**Keywords:** cones, diabetic patients, macula, risk to photoreceptors, vascular changes, fovea, cone distribution

## Abstract

**Purpose:**

Cones in diabetic patients are at risk due to metabolic and vascular changes. By imaging retinal vessel modeling at high magnification, we reduced its impact on cone distribution measurements. The retinal vessel images and retinal thickness measurements provided information about cone microenvironment.

**Methods:**

We compared cone data in 10 diabetic subjects (28–78 yr) to our published norms from 36 younger and 10 older controls. All subjects were consented and tested in a manner approved by the Indiana University Institutional Review Board, which adhered to the Declaration of Helsinki. Custom adaptive optics scanning laser ophthalmoscopy (AOSLO) was used to image cones and retinal microcirculation. We counted cones in a montage of foveal and temporal retina, using four non-contiguous samples within 0.9–7 deg that were selected for best visibility of cones and least pathology. The data were fit with a two parameter exponential model: ln(cone density) = a ^*^ microns eccentricity + b. These results were compared to retinal thickness measurements from SDOCT.

**Results:**

Diabetic cone maps were more variable than in controls and included patches, or unusually bright and dark cones, centrally and more peripherally. Model parameters and total cones within the central 14 deg of the macula differed across diabetic patients. Total cones fell into two groups: similar to normal for 5 vs. less than normal for 2 of 2 younger diabetic subjects and 3 older subjects, low but not outside the confidence limits. Diabetic subjects had all retinal vascular remodeling to varying degrees: microaneurysms; capillary thickening, thinning, or bends; and vessel elongation including capillary loops, tangles, and collaterals. Yet SD-OCT showed that no diabetic subject had a Total Retinal Thickness in any quadrant that fell outside the confidence limits for controls.

**Conclusions:**

AOSLO images pinpointed widespread retinal vascular remodeling in all diabetic eyes, but the SDOCT showed no increased retinal thickness. Cone reflectivity changes were found in all diabetic patients, but significantly low cone density in only some. These results are consistent with early changes to neural, glial, or vascular components of the retinal without significant retinal thickening due to exudation.

## Introduction

Photoreceptors are the potential victims of the harsh environment found in the diabetic retina (1). Due to their high rate of metabolism, photoreceptors not only can be reduced in number via apoptotic death in altered glucose models (2), but photoreceptors and outer retina have been reported to undergo a variety of changes and can be considered as potential culprits in diabetic retinopathy and diabetic macular edema (3, 4).

Visual function abnormalities in diabetic patients have long been documented by a wide variety of methods, including those that depend heavily upon cones or cone pathways such as color vision and multifocal electroretinography (5, 6). Foveal color vision deficits in diabetic patients are consistent with a combination of early lens changes that decrease the light focused on the retina, particularly for short wavelengths (5, 7), and failure of the foveal cones to properly guide light despite the lack of clinical evidence of macular edema (5). Both of these factors can decrease the capture of quanta, which then can decrease sensitivity, and also decrease the contrast and alter the spectra at the retina, which can lead to poorer performance on a wide variety of visual function tasks. With imaging, dark patches of cones can be spatially correlated with defects in the deeper fundus layers seen on OCT (8). Further there is neuronal damage that includes additional retinal neurons (9–12). In diabetic patients without clinically significant macular edema, there is not evidence of cones so shortened or photopigment so dilute that spectral sensitivity is altered for L- and M- cones, i.e., those that contribute substantially to foveal vision and visual acuity. Thus, while decreased color vision, particularly for short wavelengths, and poor contrast interfere with tasks of daily living in diabetic patients, biomarkers for detection and management require sensitive or specific information.

Cones underlie visual acuity, fine color vision, and fine spatial discriminations that require dense packing. Cones are irreplaceable and in humans form complex neural connections, leading to extensive signal processing in the retina. Living human cones can be individually mapped by increasing the magnification of the retinal image, using scanning to improve the contrast of the retinal image, and correcting the flawed optics of the eye with adaptive optics, in an adaptive optics scanning laser ophthalmoscope (AOSLO), with differences in techniques among laboratories (13, 14). While the number of human cones is set before birth, cones are known migrate to during the first 5 yr of life to densely pack a normal fovea (15), and become less densely packed with aging (16–18). The young fovea has a higher cone density along the horizontal meridian than the vertical one, similar to a visual streak. There are large individual differences among subjects found by several laboratories, particularly at the fovea, previously reviewed (14, 18, 19). In older subjects the horizontal vertical asymmetry is not always preserved (17). While the density of cones decreases monotonically from fovea to periphery in the healthy eye, the large interindividual differences in cone density distribution across the retina must be modeled with at least two parameters, such as overall numbers of cones and the slope of the decrease from the fovea outward (19). That is, one individual does not have a constant proportion more cones across all retinal locations than another; instead cone density at 2 and 7 deg is uncorrelated across individuals. Instead, there is a strong negative correlation between cone density at 2 deg and the ratio of cone density at 7 and 2 deg, expected because when cones migrate from the periphery to the center, then there are fewer in the periphery.

In diabetic patients without proliferative diabetic retinopathy or clinically significant macular edema, there is clear evidence of relatively intact foveal cones in humans that is provided by SD-OCT B-scans (20). However, on a finer scale, what are the potential changes to living cones and the microenvironment? Using AOSLO in the same subjects, we find that there is remodeling of retinal vasculature (20, 21). Extensive vessel remodeling occurs in some patients that is earlier than expected by clinical classification, and in some cases accompanied by numerous hard exudates or cysts too small to be seen without adaptive optics. Microaneurysms, cysts, and hard exudates smaller than the resolution of instrumentation found in the clinic, and even in the OCT images, are routinely visualized in our diabetic subjects. This both indicates a metabolic challenge to the cones, and also can make accurate quantification difficult when imaging through the overlying retinal pathology.

Despite the surprising ability of cones to survive and guide light in atrophy (18, 22) creating and maintaining high cone density in the fovea and a smooth distribution across the posterior pole may be difficult in a metabolically challenged retina. The migration of cones during early development places foveal cones in the foveal avascular zone, where even in healthy eyes there are few retinal vessels to help support photoreceptor metabolism. Inner retinal thickness is closely related to the proximity to retinal vessels, but there are large individual differences in size and shape (23). The foveal avascular zone is now rapidly and readily measured non-invasively with SD-OCT (3) and OCTA (24), again with individual variations in the size and shape, demonstrating the increased metabolic load in aging and for many diabetic foveas due to capillary closure and dropout. This indicates an even greater challenge for the retina to support foveal cones.

There is evidence that cones do not merely die in response to changes in metabolic support, but instead they can be found in highly irregular distributions. In patients with dry age-related macular degeneration there are patches of cone density at some perifoveal locations that are significantly greater than normative values, while in other locations there is atrophy with few cones (18). It is not yet known if these cones migrated slowly to regions with better metabolic support, the retina became distorted and stretched, or both. The metabolic support to maintain the arrangement of dense cones in the fovea, a visual streak, and a uniform distribution of cones, may be challenged also in diabetic patients. In young Type 1 diabetic subjects measured with AOSLO at 7 deg, eccentric to the fovea or perifovea and limited to a single eccentricity, a significantly lower number of cones is not reported, but there is a lack of the asymmetry of horizontal to vertical cone density that is the basis of the visual streak that is seen for young control subjects (25). Using the imaging device from this study and similar ones that scan to improve the contrast of retinal images is important because of contrast degradation from poor tear film, early cataract, or retinal changes that are common in diabetic eyes. This instrumentation also provided a wide field image so that the measurement locations were assured. It could be the case that the cones did not die off but never attained or could not maintain the asymmetry due to the metabolic challenge of diabetes. As the foveal peak was not measured, the potential for a meaningful proportion of fovea cones being lost was not studied.

Cone measurement results differing in some aspects were found for nearer the fovea (230–460 microns) in a study that used an AO device that lacked scanning to increase contrast and eye positioning other than pupil monitoring (26). The diabetic patients having increased central macular thicknesses indicates that diabetic changes are already measurable. At the locations measured, limited to fixed perifoveal samples, about a 10% decrease in cones is reported. Large individual differences are found, with 3 of 11 diabetic subjects having some values above the control densities. Foveal density values nearest the fovea are less than we find for young control subjects. A follow-on study reports a similar average decrease of cones in diabetic patients but only at a single eccentricity and again with large individual differences (27). To discriminate subjects with no retinopathy vs. controls also requires metrics besides cone density to quantify variability in distribution, i.e., linear dispersion and a heterogeneity packing index. Similarly, a cone heterogeneity packing index, again at only a single eccentricity in the perifoveas of diabetic subjects but with AOSLO, indicates a more disorganized cone distribution with poorer deep capillary plexus flow on OCTA (28). In a separate study using AOSLO and measurements limited to 4.6 deg eccentricity, cone density and spacing are not associated with presence of diabetes or severity of retinopathy other than presence of diabetic macular edema, but variability of cone packing is significantly associated (29). We previously showed striking changes in cone reflectivity in some diabetic eyes, with a variety of sizes of patches of dark cones that make quantification at specific locations challenging (8). Taken together, these previous studies demonstrate the potential for less regular distribution of cones in metabolically challenged eyes. However, the measurements do not clearly address the potential for decrease of cone density because single point measurements cannot be adjusted in position to avoid retinal pathology and therefore may be unable to detect small changes, have wide confidence intervals due to individual variability, suffer in some instruments from positional uncertainty, and most importantly cannot model the two factors needed to specify cone density or total numbers. Thus, a better estimate of number of cones and the resulting metabolic load due to cones is needed, especially since the overall numbers outside the central fovea are much greater despite lower density.

To document retinal health and properties of cones, SD-OCT methods have been used to measure reduction in the length of photoreceptors in diabetes (30) and aging (18), which must be distinguished from diabetes. OCT methods are useful for detecting disruption to the cone distribution and cone pathway distributions including other retinal layers. However, OCT thickness measures do not provide an accurate assessment of all the changes in cones, such as subtle decreases in cone density, since the thickness of the outer nuclear layer has been shown to increase even in the presence of a loss of cones in aging, likely due to neural remodeling and Mueller cell changes that alter the thickness of the outer nuclear layer (17).

Even without structural changes detectable by standard clinical methods such as OCT thickness metrics, patients are not free of structural changes to their retinas. In a recent study (31), we compared the frequency spectra from the FFT of the OCT B-scan of diabetic patients without retinopathy and age-matched controls. These subjects, having no retinopathy detected through clinical examination, also have no significant difference in the values for the total retinal thickness for any quadrant in the ETDRS grid, nor systematic relation with age for the total retinal thickness of any quadrant. There is a trend for the thickness between the retinal pigment epithelial/choroid border to the inner segment/outer segment junction to be thinner in the diabetic subjects, with significantly thinner values at 1 deg temporal and 3 deg nasal to the fovea for diabetic subjects. Despite these structural OCT findings, which are comparable to the healthiest eyes in the previous cones studies, the frequency analysis showed differences in the diabetic eyes vs. controls. In the deeper layers of the fundus, the diabetic patients had relatively higher amplitudes at the spatial frequencies that contained outer retinal features, capillaries, and structures of similar sizes, and less amplitude in several other frequency bands. It is not known whether neuronal or vascular changes led to the shift in frequencies present in the OCT images, but these changes indicate very early changes to diabetic eyes. Similarly, recall that the above length and thickness measurements occur within a microenvironment that we now know has extensive changes early on, compared to the retinal structure expected by clinical classification (20). Therefore, quantifying neuronal changes may provide earlier detection of eyes at risk and improve management of an individual eye or patient, as well as benefit the understanding of neural-vascular coupling, but it should not be assumed that neuronal changes occur prior to any vascular changes.

## Materials and Methods

### Subjects

We compared 10 diabetic subjects (28–78 yr, 54.7 ± 12.8 yr; seven females and 3 males) tested between 2/18 and 5/19 to our published norms for cone density in 36 younger and 10 older controls (16, 17, 19). The 36 younger normal controls were aged 18–34 yr, mean 24.4 ± 3.42 yr (19). The 10 older normal controls (16, 17) were aged 51–65 yr, mean 56.3 ± 3.71 yr. Control subjects had strict limitations on refractive error, since myopic subjects have sufficiently different cone density distributions to obscure the effects of aging or disease (32). Subjects had axial lengths that indicated a lack of high myopia or hyperopia, ranging from 22.0 to 24.1 mm (mean = 23.0 ± 0.660 mm). All subjects were consented and tested in a manner approved by the Indiana University Institutional Review Board, which adhered to the Declaration of Helsinki.

### Apparatus and Image Acquisition

Following consenting and pupil dilation, we imaged cones and retinal vasculature using a custom adaptive optics scanning laser ophthalmoscope (AOSLO), similar to previous methods (8, 16–20, 32, 33). Each patient first underwent imaging with an en face and wide field SLO, SD-OCT, and OCT-A (Heidelberg Spectralis II, Heidelberg Engineering, Heidelberg, Germany) to further document retinal status. A dense macular grid was obtained under fixation control for each eye. As previously, the wide field SLO provided a template for the AOSLO imaging. The axial length was quantified using an IOLMaster (Zeiss Meditec, Carlsbad, CA), as previously, to provide correction for sampling and computations of cones. Normative data for SD-OCT were obtained from our previous study of the analysis of retinal thickness from 33 diabetic patients without retinopathy and 33 age-and sex-matched controls, aged 39–77 yr, mean 54.1 ± 9.40 yr (31). These data provided a two-tailed, 95% confidence interval for controls, who did not differ as a group in age from the sample of diabetic subjects evaluated for cone distribution. The SD-OCT and OCT-A images were used to characterize the diabetic patients, along with a dilated fundus examination. Selected clinical characteristics and demographic information is given in Table 1. The diabetic patients ranged from two with no background retinopathy through one with proliferative diabetic retinopathy (Table 1).

**Table 1:** Age, sex, clinical characteristics, and sample results for the diabetic patients.

**Age in yr**	**Sex**	**Axial length in mm**	**DR stage**	**Features**	**CRT in microns**	***R*** **^2^**	**a**	**b**	**Total cones in 14 deg**
28	M	23.36	Bkgd DR	MA, d/b heme	316	0.908	−0.00066	10.40	175,813
39	F	22.76	Proliferative DR	NVE, peripheral PRP, venous beading	291	0.984	−0.00063	10.43	189,193
50	F	22.69	Bkgd DR	MA, d/b heme	291	0.893	−0.00058	10.77	282,972
56	F	22.17	No Bkgd DR		246	0.873	−0.00039	10.45	263,505
56	M	24.10	Bkgd DR	MA only	261	0.946	−0.00060	10.31	174,847
58	F	23.05	Severe non-proliferative DR	Venous beading, d/b heme	246	0.924	−0.00060	10.37	185,808
59	F	23.84	Bkgd DR	MA, d/b heme	261	0.851	−0.00034	10.28	240,853
61	F	21.99	Bkgd DR	MA only	281	0.791	−0.00068	10.83	265,231
62	F	22.38	No Bkgd DR	RPE lesion	267	0.856	−0.00043	10.54	272,577
78	M	23.28	Bdgd DR	MA only	265	0.767	−0.00062	10.32	172,712

*DR stage, diabetic retinopathy stage; CRT, central retinal thickness; R^2^, linear fit to exponential model; Bkgd DR, background diabetic retinopathy; MA, microaneurysms; d/b heme, dot blot hemorrhage; NVE, new vessels elsewhere; PRP, pan retinal photocoagulation; RPE, retinal pigment epithelium*.

In the custom AOSLO, two imaging beams were simultaneously delivered (769 and 842 nm) with 160 and 110 microwatts at the pupil, respectively, using a supercontinuum laser (NKT Photonics, Birkerod, Denmark). For the present quantification of cones, confocal images with an aperture of 100 microns at the detector (two Airy disks) were analyzed from the data of the optical channel with the 769 nm imaging beam. The illumination was scanned in a raster pattern with a horizontal frequency of 15.1 kHz and a vertical frequency of 29 Hz to produce 1.9° × 1.6° (W × H) retinal images of 780 ×520 pixels. All images shown were acquired at a constant magnification that is practical for these large datasets, but even better resolution of the very narrow foveal cones is also collected at higher magnification (not shown). Each subject's optical aberrations were measured at 20 Hz using a Shack-Hartmann sensor that allowed for correction over a customized size and shape of pupil (34). This optimized the optical correction on an individualized basis and allowed AOSLO imaging in subjects who had pupils less than the full 8 mm or mild lens changes.

**Figure 1:**
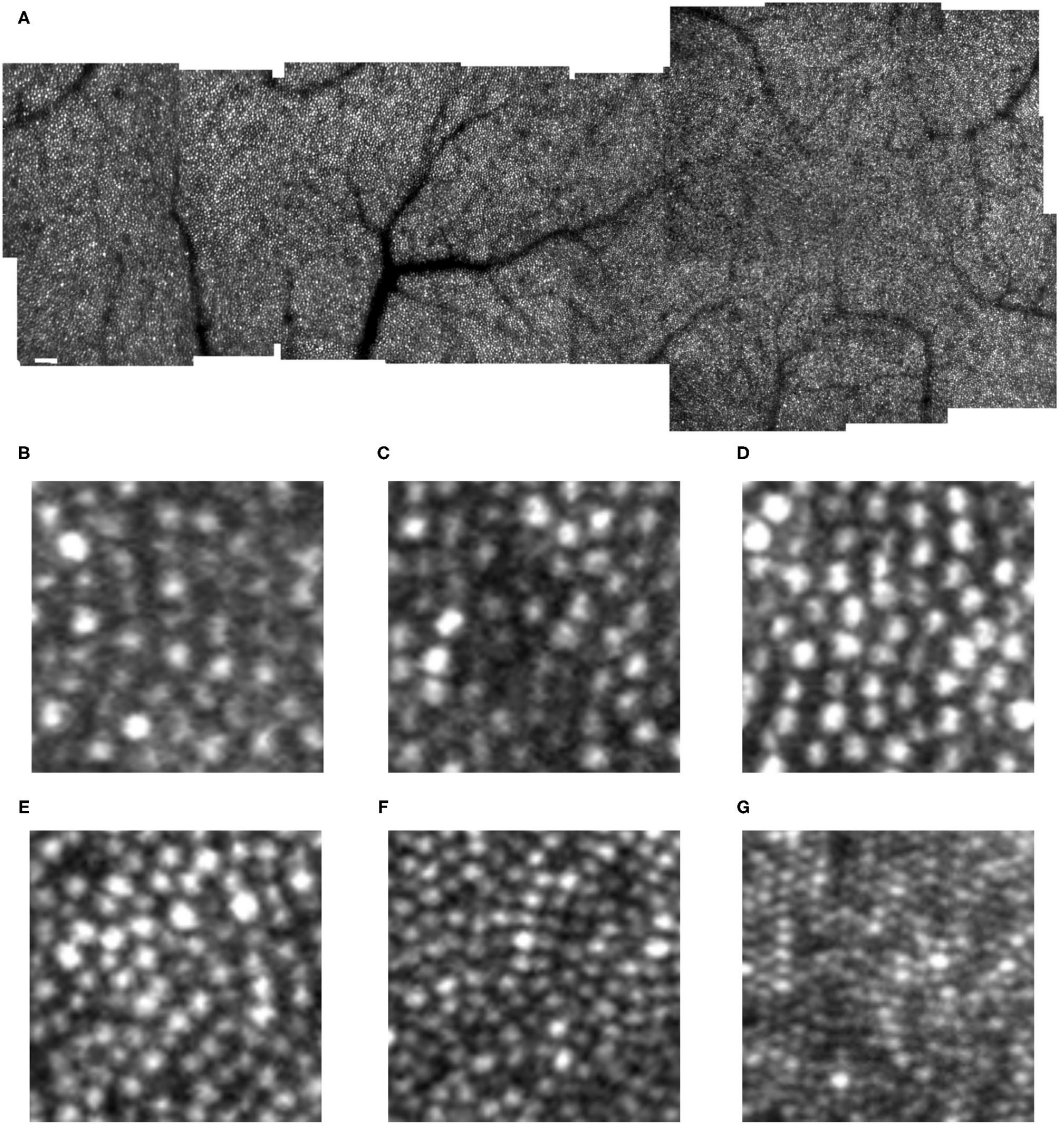
Cone distribution for a 25 yr old female control subject, shown in a montage of individual images **(A)** of the right eye collected with the confocal mode of the AOSLO. The fovea, which is on the right, has the highest cone density. There is smoothly decreasing cone density farther from the fovea, with the temporal meridian shown. Small white scale bar at lower left = 100 microns. **(B–F)** Individual cones shown at increased magnification in regions of 100 × 100 microns with the upper far corner at 8.7 **(B)**, 8.4 **(C)**, 7.0 **(D)**, 4.8 **(E)**, 2.8 **(F)** temporal and 1.1 deg temporal inferior **(G)** deg temporal with respect to the fovea.

Typically 100 frames of video were collected for each image acquisition, using a graphics overlay on the en face SLO image to steer the image capture to the approximate position as the subject looked straight ahead. For all subjects tested that provided normative values for younger and older subjects, the samples formed a “+” shape around the fovea to include not only temporal but also inferior, superior, and nasal meridians to at least 7 deg eccentricity (16, 17). For later subjects (8, 18, 19), an abbreviated protocol was used for most subjects to ensure the highest quality data (Figure 1). This shorter session reduced the decrease in contrast found particularly in older subjects due to tear film irregularities increasing over time within an imaging session. The temporal meridian was chosen for emphasis because there are fewer overlying blood vessels to interfere with sampling. Further, individual differences in the peripapillary region that can be large in older subjects were therefore not a factor.

Following imaging of cones, the focus of the AOSLO was set more superficially, and imaging of the retinal vasculature in the foveal and temporal regions of interest was performed using both channels. These data, along with the SLO and OCT imaging, helped document dilated capillaries, small hemorrhages, and microvascular remodeling, needed to guide the sampling of cones away from artifacts. These portions of the protocol for experimental imaging techniques with AO typically required about 40 min for the 90 subjects tested to date, exclusive of ophthalmic exam and detailed results discussions with patients.

### Image Analysis

After removing the sinusoidal scanning pattern motion, corrected images were processed by automatically detecting a frame without significant eye motion and then aligning other frames within the 100 frame sequence to the template image using a strip alignment method, as previously described (8, 19). Frames with large eye motions or blinks were automatically rejected by the computations. To improve contrast of summary images for grading, the lucky average images were typically used for cone counting (35). The average data for each sampled location were built into a montage, with about 50% overlap between samples, and additional overlap when acquiring samples left to right vs. right to left. The montage method eliminates the need for precise fixation by the subject to determine locations for sampling, as long as fixation is stable enough for averaging.

To count cones, we used the software developed previously that provided the younger and older normative data (16, 17, 19) and cone counts in diseased retinas (18), with upgrades to display more channels and choices of display contrasts and magnifications (MATLAB, Mathworks Inc, Natick, MA). We sampled temporal retina, ~0.9 to 7 deg to obtain 4 non-contiguous samples of roughly 100 × 100 microns. Many diabetic datasets contained overlying retinal vascular lesions, but the software allowed selection for best visibility of cones and least pathology, with the eccentricity corrected for axial length. Spacing the samples apart and not oversampling at the fovea provide the best comparison of patient to control data, since the data near the foveal center are the most variable and some subjects have a steeper decrease of cones near the fovea than others (19).

### Cone Distribution Model

The data were fit with a 2 parameter exponential model:


(1) $$\begin{array}{r} \ln\left(cone\;density\right)=a*microns\;eccentricity+b \end{array}$$

where a is a slope parameter that indicates the steepness of decrease in cone density from fovea to periphery and b is an offset parameter. As b is weighted heavily by points outside the fovea in our typical sample of four spaced positions, then b is related to an asymptotic density of cones in the periphery and the overall number of cones within the error of the fits. As previously discussed, the fits of the exponential model yield excellent *R*^2^ values when there are four widely spaced data points, but some subjects have a steeper slope near the fovea than is described by an exponential model (19). Further errors occur when there are large variations in the density of cones in association with diseased retinas (18).

Total cones within the central 14 deg of the macular were estimated from parameters a and b, as previously (19). The previous data were normalized for the horizontal:vertical asymmetry in cone density. Given the lack of this asymmetry in several of our older control subjects (17) and potentially in diabetic patients (25), we compared the temporal quadrant cone model without further normalization.

### Retinal Thickness From SD-OCT

Vendor supplied software provided the mean and SD for each subject for total retinal thickness of quadrants within a standard ETDRS grid. The center region of interest is a foveal center circle that is 1 mm wide with respect to the emmetropic retina. The inner nasal, superior, temporal, and inferior quadrants cover from 1 to 3 mm. The outer nasal, superior, temporal, and inferior quadrants cover from 3 to 6 mm. The normative data are those previously reported for our institution (31).

### Statistical Analysis

The density of cones data were computed with custom software, as described previously (17–19). The fits to the exponential model were performed by using linear regression on the ln(cone density) as described previously (19), and the confidence limits were computed, using Excel and Statview. The total cones modeling was performed in Excel as described previously (19). The Total Retinal Thickness means and standard deviations were computed from SD-OCT, using vendor software on the Spectralis and then compared via ANOVA with SAS and with linear regression with Statview.

## Results

### Cone Appearance in Diabetic Maculas

Cones could be counted in all subjects. In the young, healthy eye, there is a systematic distribution of cones, which provides an organized sensor array with the highest cone density at the fovea and cone density decreasing sharply with increasing distance from the fovea (Figure 1). In the young, normal eye there is a wide variation in the reflectivity of individual cones, but primarily a regular pattern of reflectivity differences of single bright or dark cones. Regions of darker appearing cones beyond a single cone are typically in the shadow of overlying retinal blood vessels. Cones increase in size and center-to-center spacing, on average, with increasing eccentricity (Figures 1B–G). Small disruptions in the cone mosaic occur in normal subjects and are intentionally shown (Figures 1E,F), along with individual cones that are not uniformly guiding light Figures 1B,C). It is typical that disruptions with age are more common (17), but not as extreme as those found in subjects with atrophy (18). Even in control subjects, overlying retinal vessels can shadow cones (Figure 1F) and make it more difficult to count them.

**Figure 2:**
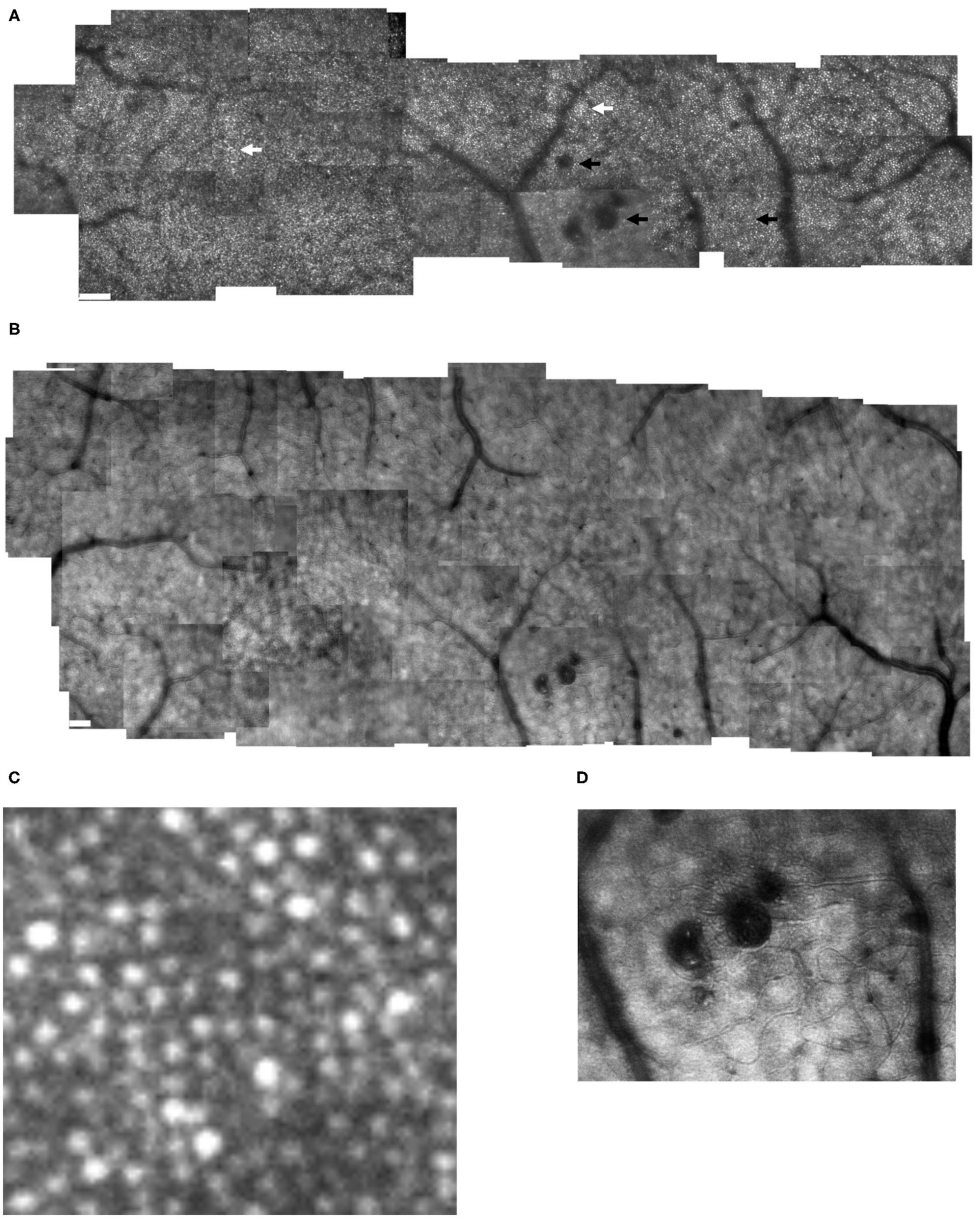
Cone distribution for a 59 yr old female diabetic subject, collected with the confocal mode of the AOSLO **(A)**, shown in a montage of cone images with the foveal region on the left. The retinal microvasculature layer montage **(B)** shows a strong response to ischemia, with numerous retinal vascular changes shadowing the cone layer: multiple microaneurysms with vascular remodeling surrounding them, capillary bends or loops, and doubled or collateral vessels, particularly near microaneurysms or complex tangles. The montage of individual images shows that cone density is highest in the fovea, then decreases with increasing eccentricity, with single cones or patches of cones being unusually bright (white arrows), as well as patches of dark cones. There are regions in which the overlying retinal vessel remodeling interferes with measurements of cones, by obscuring them (top black arrow). Sometimes the contrast across an entire individual image sequence of cone images was decreased, either due to the tear film or a decreased the wavefront correction across in a large area of edema (bottom left black arrow). Elsewhere, the contrast is decreased for regions smaller than an entire image, which rules out anterior segment artifact as the sole source of image degradation (bottom right arrow). Small white scale bars at lower left = 100 microns. A magnified region of 100 × 100 microns **(C)** shows patches of bright cones and areas of decreased contrast. An enlargement of the retinal microvascular layer **(D)** shows microaneurysms and extensive vessel remodeling. The cone parameters were a = −0.00034, and b = 10.28, outside of normal limits. RMS fit = 0.851.

**Figure 3:**
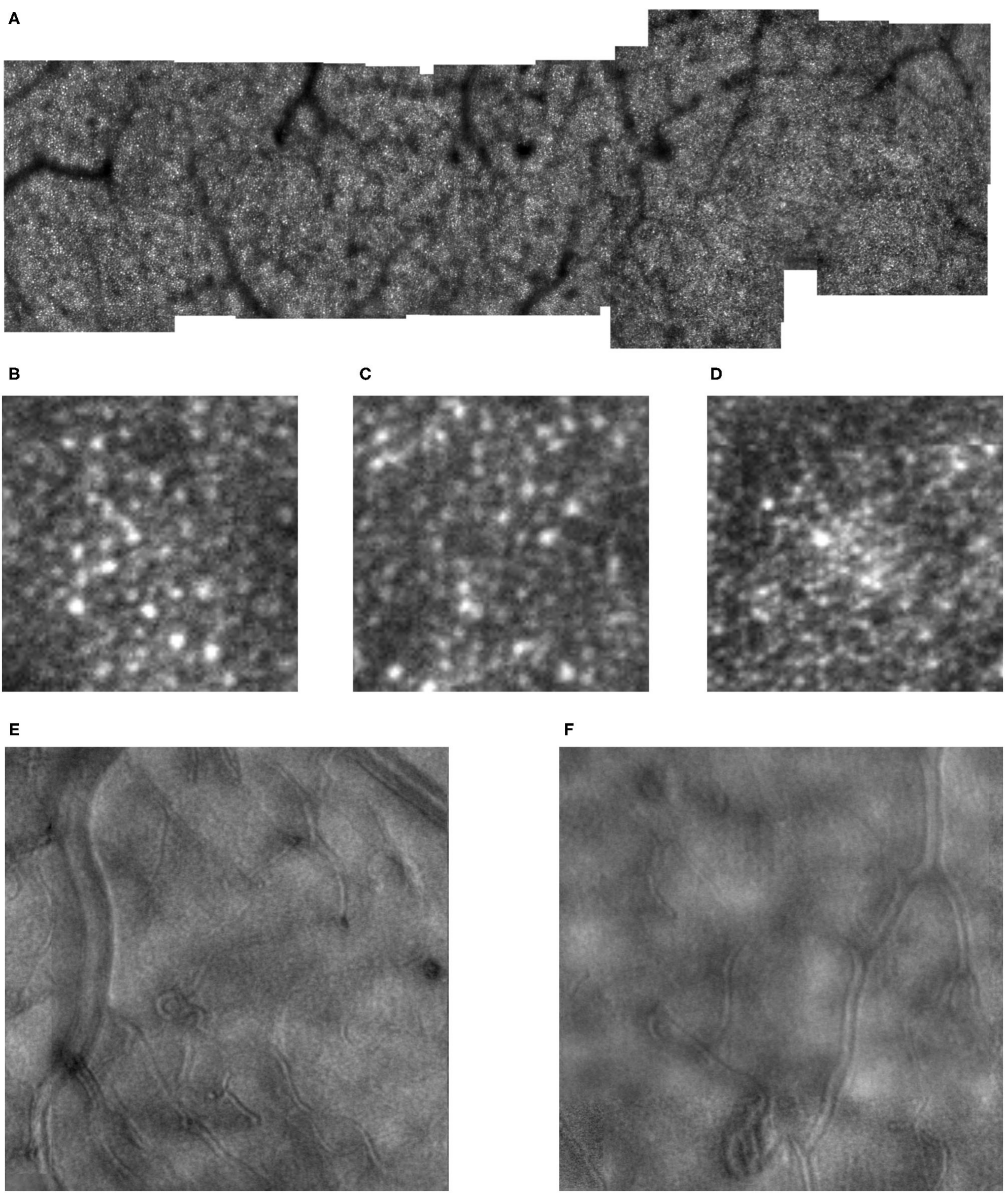
Cone distribution for a 61 yr old female diabetic subject **(A)**, shown in a montage of cone images with the foveal region on the right, with a more moderate response to ischemia than in Figure 2. Small white scale bar at lower left = 100 microns. There are microaneurysms, but less frequent capillary bends or loops, and few doubled or collateral vessels or the more complex tangles. **(B–D)** Magnified view of cones, showing the phenomena of both extra bright and dark cones in unusually large numbers in 100 × 100 micron samples of cones, with the upper far corner at 7.6, 5.6, and 1.0 deg temporal with respect to the fovea. **(E,F)** Enlarged views of the retinal microvascular layer, showing 500 × 500 microns samples of microaneurysms, capillary bends, and elongated and collateral vessels, but smaller or less frequent than in Figure 2. Cone parameters, were a = −0.00068, and b = 10.83, within normal limits. RMS fit = 0.791.

While previous cone distribution modeling resulted from sampling regions of interest at fixed locations, we found that in all diabetic subjects there were retinal vascular changes and obscuration from retinal remodeling that required moving the sampled regions of interest to locations that were not at the precise eccentricities of the control data (Figures 2, 3). At some locations, obvious shadowing due to overlying retinal vessels or retinal remodeling prevented sampling. Thus, the amount of displacement of the sampled region of interest was considerably greater in diabetic patients than the minor changes required in sampling the control data. There were scattered low contrast regions, larger than in older controls but smaller than the AOSLO image. Patchiness of the clarity of focal regions in the images of diabetic eyes was not due to variation in image quality related to optical degradation of the anterior segment, as this would alter the clarity of the whole sampled image at once. That is, pupil plane artifacts lead to roughly uniform degradation of contrast in the retinal image of an AOSLO. However, there were also occasional pupil plane artifacts, evidenced by an entire image sample within the montage being of lower contrast, indicating fluctuations in correction by the AO over time, such as with variations in tear film. Thus, although for our control subjects, and most previous studies, sampling was at roughly the same retinal eccentricities along a temporal meridian, for the diabetic subjects we fit the exponential model to each subject's data to obtain parameters for cone density as a function of retinal eccentricity.

Diabetic cone maps showed that cones were found distributed over a wide variety of locations, but included bright patches, with unusually bright cones alone or in clusters, and clusters of dark cones as noted previously (Figures 2, 3). These unusually bright or dark cones were not limited to the fovea and perifovea. Similarly, over the entire montaged region there is a qualitative disruption to a smooth distribution of cone density, previously noted for the perifoveal region. These two findings indicate that disruption to the cone distribution is not limited to just locations near the foveal avascular zone.

### Cone Distribution and Estimate of Total Cones in Diabetic Maculas

**Figure 4:**
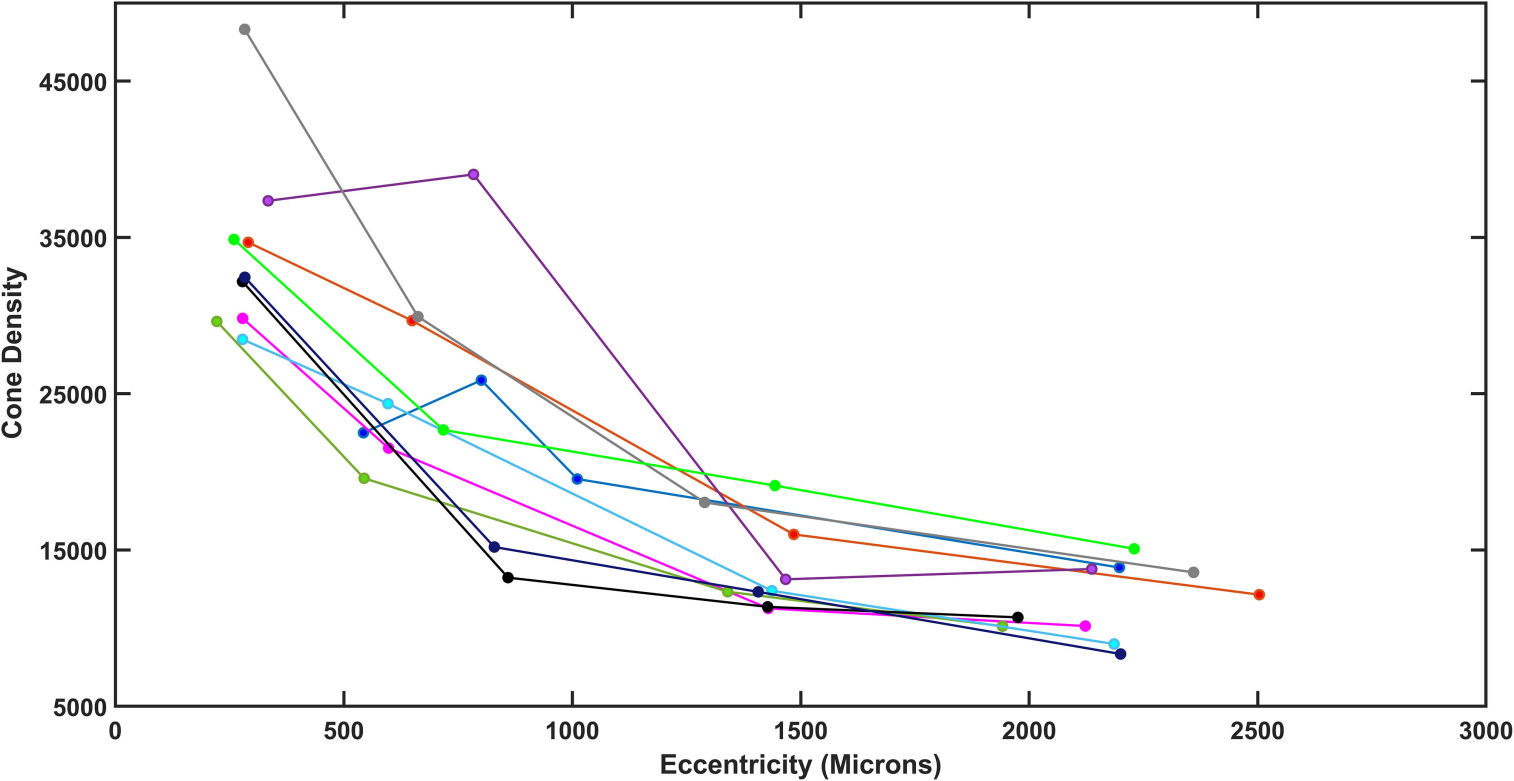
Total density plotted as a function of retinal eccentricity for the diabetic subjects, showing differences among subjects at all eccentricities.

As humans differ in both the overall numbers of cones and the steepness of the decline in cone density from fovea to periphery, determining cone loss from a single location can be misleading. The normative data indicate a monotonic decrease in cone density from the fovea to greater eccentricities. High cone densities are more typical in younger subjects, which correspond in some subjects to a steeper decline in cone density with increasing distance from the fovea, so that more peripheral cone densities in the periphery overlap to a greater extent than near the fovea. The decrease in cone density for diabetic subjects also varied across subjects, not just in the overall numbers of cones but also the rate of decline (Figure 4). While there was a general decline in cone density from the fovea toward the periphery, some subjects had densities that did not decline monotonically due to local variability.

**Figure 5:**
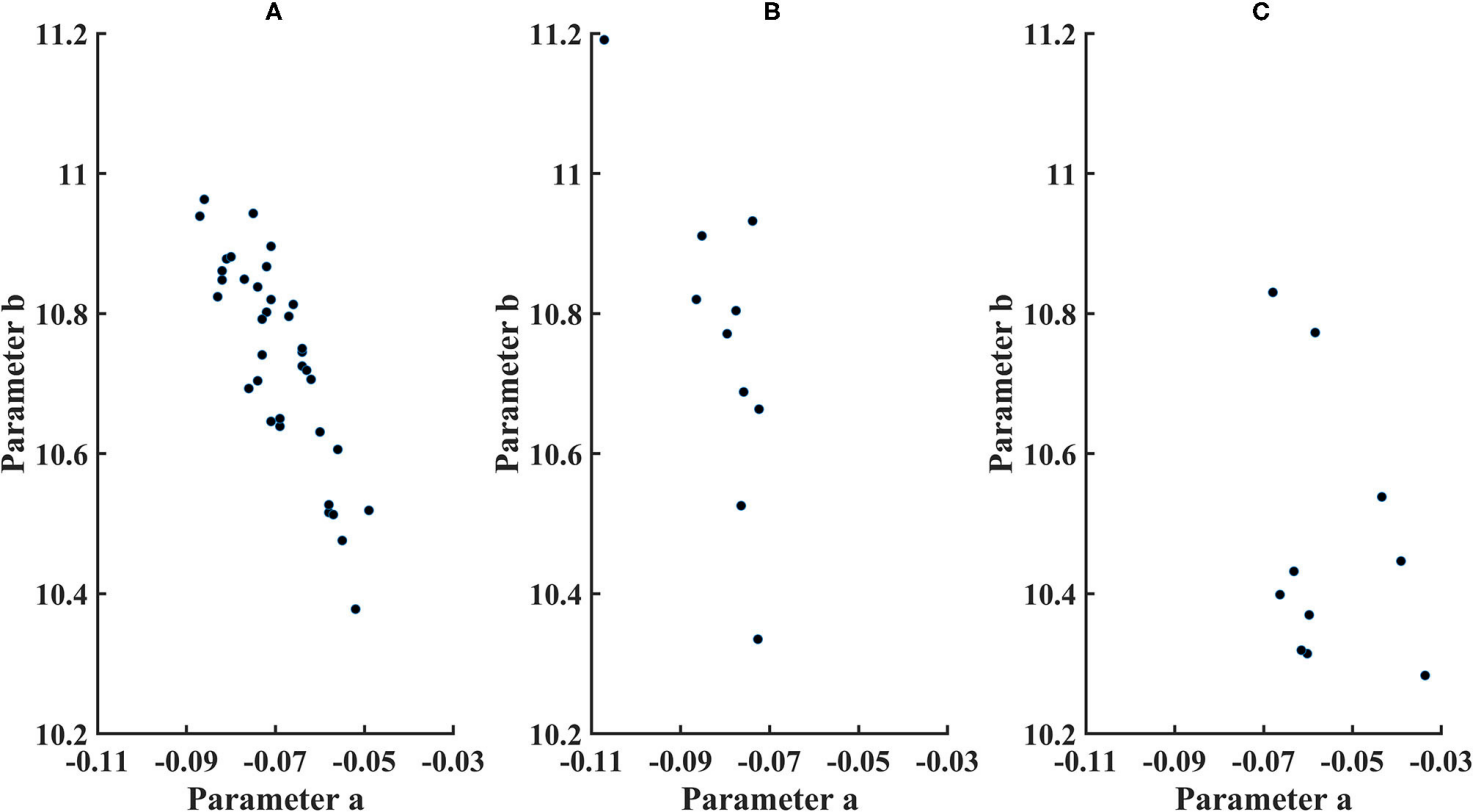
Parameters for cone distribution from the exponential model ln (*cone density*)=a*microns eccentricity+b, with a plotted as a*100. **(A)** younger control subjects, **(B)** older control subjects, and **(C)** diabetic subjects.

The exponential model provided excellent fits for both younger and older controls: for the linear fit to the ln density transform for four widely spaced samples, *R*^2^ = 0.9371 ± 0.0387 and 0.959 ± 0.0267, for younger and older, respectively (Figure 5). Thus, while the variability for older subjects in cone density is large, the goodness of fit indicates that this is an overall phenomenon and not due to local variability. Inspection of the cone maps on and between samples supports this conclusion (17). The fit to the model was similarly good for some diabetic patients and poorer for others. The *R*^2^ was 0.767–0.984 in diabetic data set. Despite the large proportion of variance accounted for in the diabetic patients, the control data fit the model so well that four of 10 diabetics had *R*^2^ < the 95% confidence limit for younger or older controls, as appropriate. Thus, while the model fit the data sufficiently well to determine whether the diabetic patients had a less peaked foveal slope or fewer overall cones, as compared to age-matched controls, the variability is consistent with a less even distribution of cones in diabetic eyes, as previously reported for small perifoveal regions. The variability is seen when examining the cone maps of diabetic subjects (Figures 2, 3).

The 95% CI's were computed from the exponential model. Parameter b (younger subjects) = 10.7 ± 0.146, with the 95% CI = 10.4–10.9. Parameter a (younger subjects) = −0.000692 ± 0.0000985, with the 95% CI = −0.00050 to −0.000885. Parameter b (older subjects) = 10.7 ± 0.222, with 95% the CI = 10.4–11.3. Parameter a (older subjects) = −0.0008071 ± 0.0000984, with the 95% CI = −0.000582 to −0.00103. For the older subjects, three of 10 subjects had lower values of parameter b than younger subjects, and one of 10 subjects had a lower parameter b than the confidence limits for younger subjects. For parameter a, there was considerable overlap between younger and older subjects, with great variability for older subjects but only one of 10 older subjects falling outside the confidence limits for younger subjects.

Overall cone density was often lower in diabetic patients, as indicated by parameter b compared to the lower 95% confidence limits (Figure 5). For the two younger diabetic subjects, parameter b fell below the 95% confidence limit for younger controls. For five of the eight older diabetic subjects, parameter b fell below the 95 % confidence limit for older controls, for a total of seven of 10 diabetic subjects having low values for parameter b. If parameter b is thought as analogous to an asymptote for cone density at the greater eccentricities, then the data can be examined for trends at the greater eccentricities measured, i.e., the cone density that a metabolically challenged retina could support. The fit is consistent with five of the 10 subjects having cone densities at the greater eccentricities that clearly fell below the densities other five (Figure 4). Further, there is a range of cone density among the top five subjects with two subjects falling below the other three.

The decline in cone density from the fovea outward was often shallow in diabetic patients, as indicated by parameter a. All diabetic patients had slopes less than the mean for their age group. For the two younger subjects, parameter a was not below the 95% confidence limit. For the older diabetic subjects, three of eight had slopes shallower than the 95% confidence limits for their age group.

**Figure 6:**
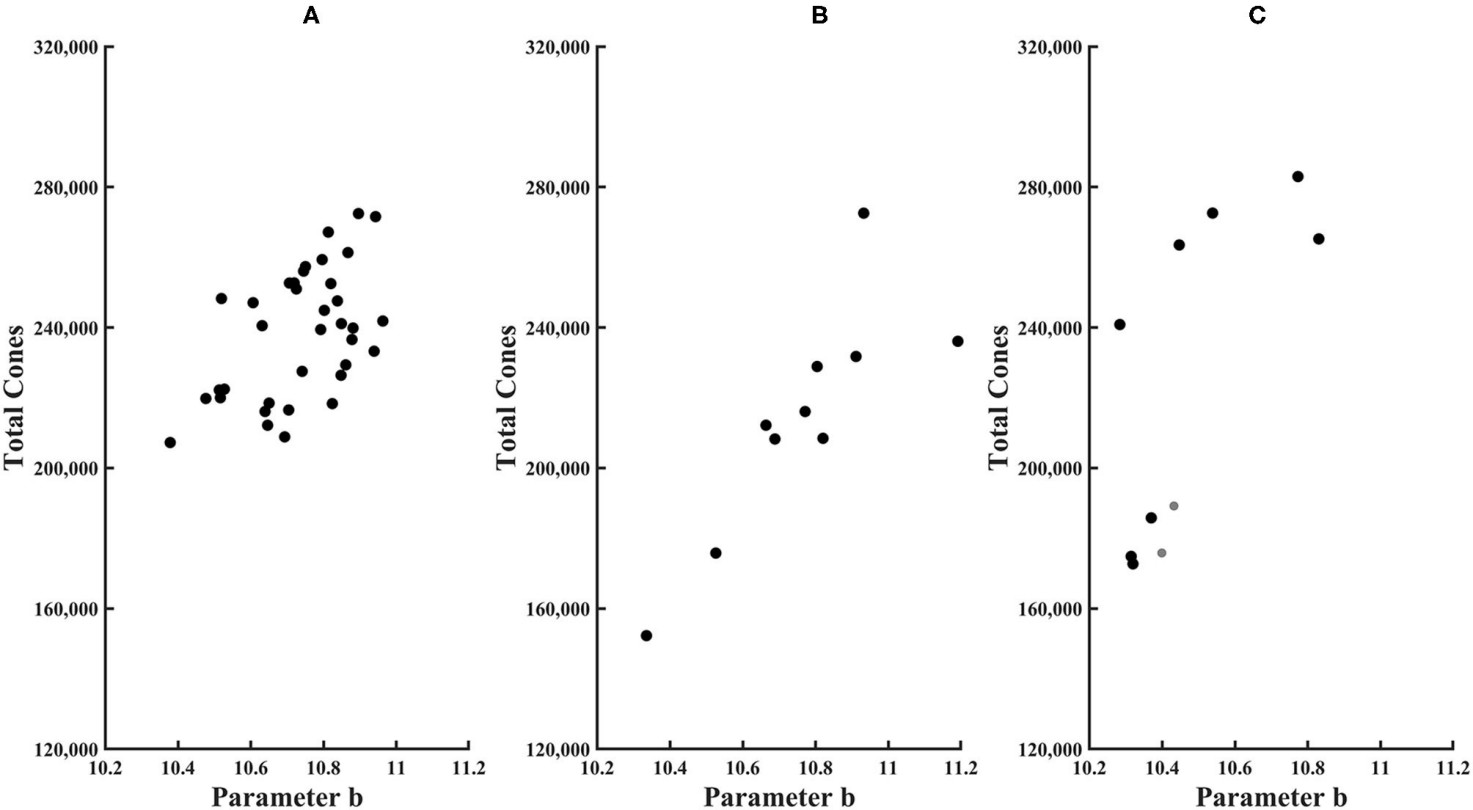
Total cones plotted as a function of parameter b of the exponential model for **(A)** younger control subjects, **(B)** older control subjects, and **(C)** diabetic subjects. Younger subjects have smaller symbols.

Total cones in the central 14 deg of the macula, as estimated from temporal quadrant data, were 238,000 ± 18,300 for younger subjects, with a minimum of 207,000 and a maximum of 272,000 (Figure 6). The 95% confidence limits were 203,000 and 274,000. Total cones for the 10 older subjects were 214,000 ± 33,000, resulting in wide confidence intervals: a minimum of 140,000 and a maximum of 289,000. Total cones were lower than the mean of the younger subjects in nine of 10 older subjects, statistically different from chance (Binomial test with 10 df, *p* = 0.00977). However, only two of eight older subjects had total cones less than the lower 95% confidence limit of the younger subjects. There were large individual differences in both younger and older control groups, as expected from the cone density data (16, 17, 19). The coefficient of variation of total cones was significantly greater for older subjects than for younger ones, 0.145 and 0.0767, respectively, [F_(35,9)_ = 4.03, *p* < 0.025]. This finding is based on, and consistent with, the high intra-individual variability of cone density reported previously in the younger subjects based on 10 of the 36 younger subjects and the same older subjects (17) and in 36 younger subjects (19). However, the greater variability of the older subjects may be in part due to a smaller sample size. While the clinical classification of a patient and the total cones were not always consistent, it should be noted that the two subjects with no background diabetic retinopathy had among the highest total cones (Table 1).

Total cones were lower in some in diabetic subjects. For both younger diabetic subjects, the total cone values of 176,000 and 189,000 fell below the 95% confidence limit. In three of eight older diabetic subjects, total cones was lower than the mean for older controls. However, given the high variability of older controls, none of the diabetic subjects had total cones either greater than or less than the 95% confidence limits of older subjects. For these diabetic subjects, there was no significant association with age for total cones, parameter a, or parameter b, with *R*^2^ from the linear regression being only 0.026, 0.070, and 0.0020, respectively.

### Structural Changes Associated With Cones in Diabetic Maculas

All diabetic subjects had retinal microvascular changes seen on AOSLO, likely in response to ischemia (Figures 2B,D, 3E,F), but to varying amounts. The strongest response to ischemia included multiple microaneurysms with vascular remodeling surrounding them, capillary bends or loops, and doubled or collateral vessels, particularly near microaneurysms or complex tangles.

**Figure 7:**
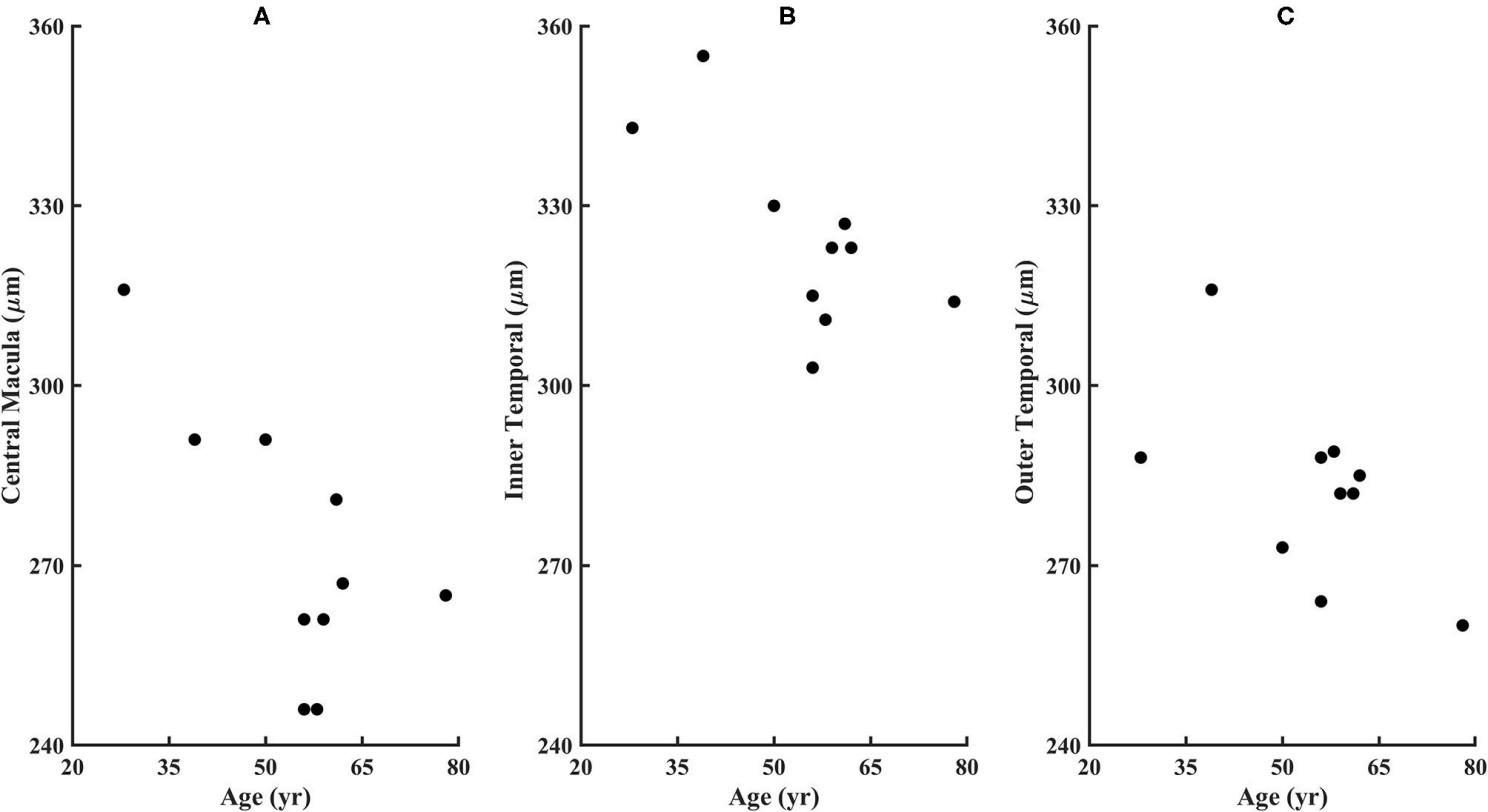
Retinal thicknesses of the diabetic subjects from the ETDRS grid from the SD-OCT for **(A)** Central Macular Thickness, **(B)** Inner Temporal Thickness, and **(C)** Outer Temporal Thickness. No subject fell outside the confidence limits from the age-similar controls, although subject 209 is missing the outer nasal thickness measurement due to fixation error. The Total Retinal Thickness is significantly correlated with age for the diabetic subjects, with *P* < 0.02, 0.02, and 0.05 for the Central Macular Thickness, Inner Temporal Thickness, and Outer Temporal Thickness.

**Figure 8:**
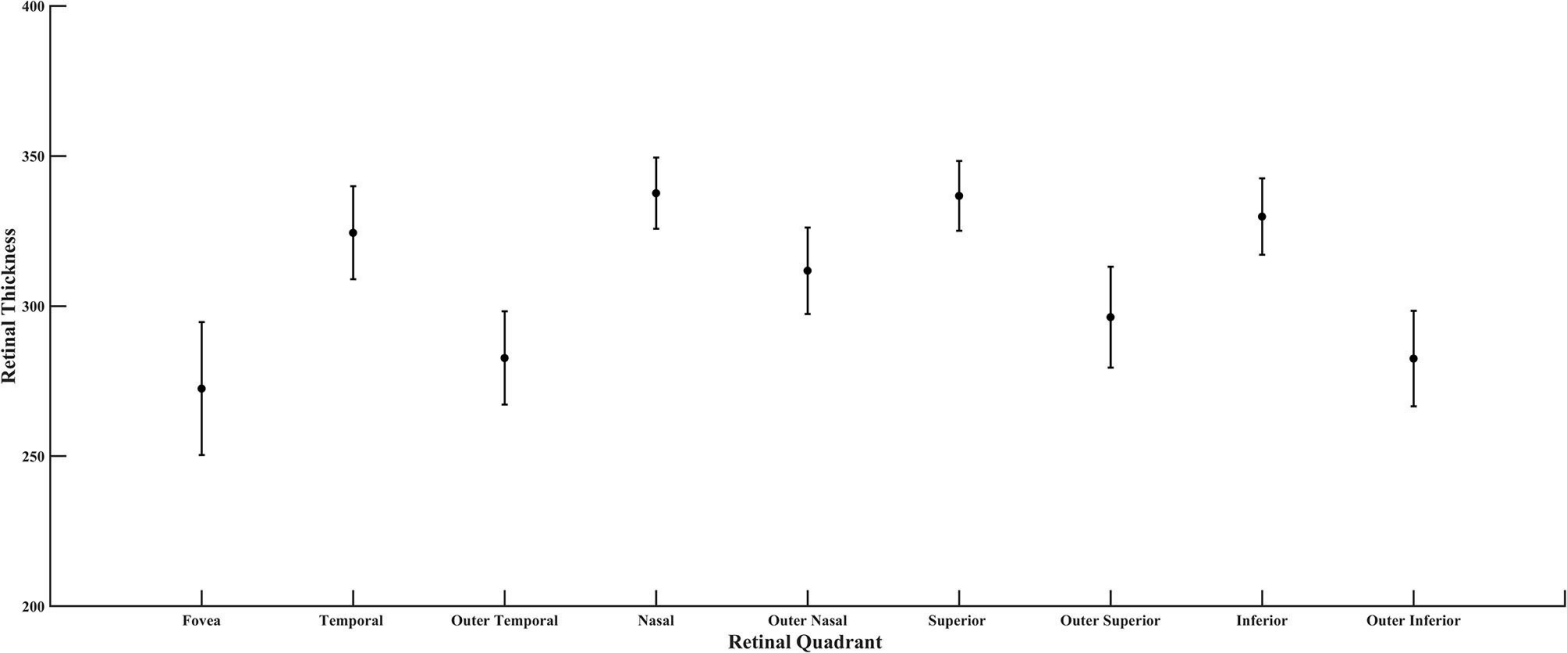
Retinal thickness of the diabetic subjects for all quadrants of the ETDRS grid, plotted as mean and standard deviation.

From the SD-OCT data for the control subjects, the Total Retinal Thickness of the three ETDRS quadrants that corresponded to cone distribution data, Central Macular Thickness, Inner Temporal Thickness, and Outer Temporal Thickness, had 95% confidence limits for the age-similar control subjects of 226–327, 290–370, and 253–322 microns, respectively. For the diabetic subjects, the Total Retinal Thickness for Central Macular Thickness, Inner Temporal Thickness, and Outer Temporal Thickness ranged from 246 to 316, 303 to 355, and 260 to 316 microns, respectively. Despite clear-cut abnormal retinal vasculature on AOSLO in all 10 diabetic subjects, the Total Retinal Thickness values for each subject in each quadrant were within the 95% confidence values, being neither thicker nor thinner than controls (Figure 7). For the other ETDRS subfields, again Total Retinal Thickness values for each of the 10 diabetic subjects were within the 95% confidence values, other than a missing Outer Nasal value for one subject (Figure 8). However, there was a significant correlation of age with Total Retinal Thickness for most of the subfields, with R = 0.700, 0.705, 0.600, 0,772, 0.390, 0.787, 0.587, 0.627, and 0.293 for the foveal, temporal, outer temporal, nasal, outer nasal, superior, outer superior, inferior, and outer inferior quadrants. Decrease in Total Retinal Thickness per year with age was 1.14, 0.806, 0.688, 0.677, 0.392, 0.677, 0.727, 0.591, and 0.345 microns, respectively. Significance of the association of Total Retinal Thickness with age varied across subfields for the diabetic subjects, with *P* < 0.005 for nasal and superior; *P* < 0.02 for foveal and inner temporal; and *P* < 0.05 for outer temporal, outer superior, and inferior; but only *P* < 0.15 for the outer nasal and *P* < 0.7 for the outer inferior quadrants. These results are consistent with relatively greater loss in Total Retinal Thickness near the fovea. In contrast to our previous results that indicated no change with age, but included only subjects without retinopathy on clinical examination (31), the present subjects had clear cut retinopathy and vascular remodeling. The lack of a measurable decrease in thickness compared with controls in the present study, but yet a significant decline with age in retinal thickness while the values fell within the confidence limits, supported the conclusions that there is a loss of neural, vascular, or glial elements.

### Cone Reflectivity and Distribution Compared for Younger, Older, and Diabetic Maculas

**Figure 9:**
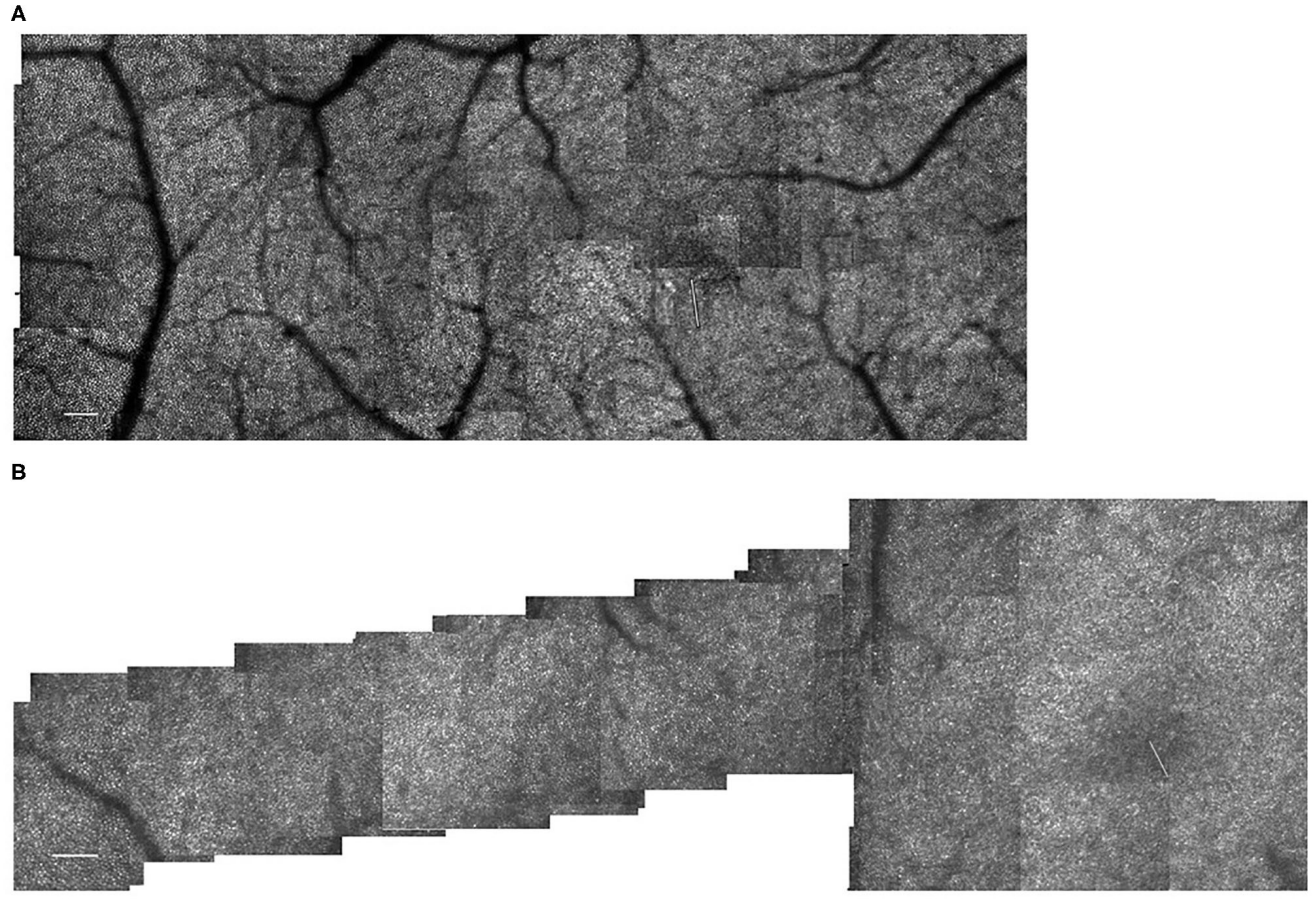
**(A)** Cone distribution for a 55 yr old female subject from our control population, shown in a montage of cone images with the foveal region on the right. This subject was selected because she had the second lowest cone densities in the sample. Small white scale bar at lower left = 100 microns. White line on the right indicates the foveal center, where the cones are not readily distinguished with this setting on the AOSLO. **(B)** Cone distribution for a 58 yr old male subject from our control population, shown in a montage of cone images with the foveal region on the right. This subject was selected because he had the highest cone densities in the sample. This subject lacks a foveal avascular zone, having readily imaged capillaries through the foveal center. Small white scale bar at lower left = 100 microns. White line on the right indicates the foveal center, where the cones are not readily distinguished with this setting on the AOSLO.

The most obvious difference between cone montages for younger (Figure 1) and older controls (Figures 9A,B) as opposed to diabetic patients (Figures 2, 3) is the lack of shadowing of the cones by overlying remodeled capillaries and microaneurysms in the control subjects. While there is shadowing by overlying retinal vessels, it is less and does not prohibit measurements. Cones in all maculas in this study varied over space in reflectivity and distribution. In all subjects, the central macula has a higher density of cones than more peripherally. However, the decrease in cone density with increasing eccentricity was monotonic in all subjects except two (Figure 4), but could be steep or gradual and overall not a large change. Older subjects have more variable cones densities, particularly in the fovea (16, 17), but this may not be obvious from viewing the montages; the two older subjects shown have the highest and the second lowest cones in the control sample. The large variation in cone density across subjects is clear from the wide confidence limits, and model parameters a and b. As previously described, there are not large areas of missing cones in normal older subjects. Although the outer nuclear layer is on average thicker in the older subjects (17), and this layer overlies the outer segments, the return of light to the AOSLO is sufficient to quantify the cones. All subjects have cones of variable reflectivity, i.e., brighter appearing cones and darker appearing cones, but the extremes are typically not contiguous in the older eyes, even in older subjects (Figures 9A,B). (In contrast, some diabetic subjects have numerous localized dark cone regions with knife edge borders that take up the space required for multiple cones (Figures 2, 3), as found previously (8). There are cones that appear much brighter than surrounding cones in all eyes, but in diabetic eyes some of these cones appear even brighter and larger than in control eyes.

## Discussion

Combining the appearance of cones, parametric data, and the model of total cones, there is evidence that cones undergo changes despite the lack of sufficient structural changes to result in thickened or thinned Total Retinal Thickness. Cones are altered in their ability to interact with light, in that there are patches of both light and dark cones. As the visualization of bright cones is via the light they return to the AOSLO, there is more than one possible interpretation of the bright cones that appear large. There is evidence for a decrease in cone numbers, which would allow cones to expand laterally. Rods were not measured, and if decreased could provide extra expansion space, but bright cones are seen in areas within the foveal avascular zone where rods are normally few in numbers (Figure 3D). Alternatively, and not mutually exclusively, cones may be leaking light, i.e., not guiding light back to the instrument is as narrow an angle or through the entire length down to the retinal pigment epithelium. This is consistent with the appearance of dark cones, also, in which leakage of light may lead to failure to guide light back to the instrument. The configuration of the custom AOSLO used here does employ a confocal aperture, but the diameter of the aperture is wide enough to allow for the capture of some multiply scattered light, trading off directionality of signal with signal intensity. Thus, we can image dark cones in the fovea (8). In contrast, an AOSLO can be configured to provide the maximum contrast from cones that do guide light, but this configuration reduces the signal from multiply scattered light or background signal, and makes difficult the detection of dark cones in the fovea due to a lack of rods surrounding the cones to provide contrast (36). The present configuration also allows some light from additional planes to be included in the signal, so that multiply scattered light from deeper or more superficial layers is detected. Thus, the early retinal damage that changes the amplitude of the frequency spectra in OCT, but is not readily pinpointed as visible features (31), can increase the brightness of the image focally. This may contribute to the increased brightness in regions with multiple and contiguous bright cones.

Several aspects of the data indicate damage in and near the fovea, as well as in the periphery. In several subjects, there was a lack of a peak maximum of cone density near the fovea or only a moderately steep decrease of cone density from fovea to periphery. The decrease from central to more peripheral locations was not always monotonic. The greatest decline with age in Total Retinal Thickness was in the fovea, with inner quadrants having more decline with age than the outer ones. While dark cones and the swollen appearing bright cones are not necessarily more numerous in the foveal region, this region is not spared of these changes.

There is evidence that the cone distribution across the retina changes in relation to diabetes, rather than just death of cones. The heterogeneity of cone spacing just outside the foveal avascular zone is a promising metric for neuronal involvement. Increased cone disarray has been shown in three studies, in comparison to controls and related to vascular changes (28) and retinal thickening on OCT (27, 29), but results were mixed with regard to whether cones decrease in numbers or migrate. We found evidence of cone reduction in some eyes on measurements not limited to locations near the fovea avascular zone, and cone distribution changes that are both qualitatively seen on montages and quantified by parameter changes in the exponential model. Further, the cone changes occurred without increases or decreases in retinal thickness on OCT. However, it cannot be determined with the cone changes began prior to any vascular changes, since there was evidence of retinal vascular remodeling in response to ischemia in all diabetic subjects. Cones persist despite clear-cut evidence of an ischemic response. Sufficient numbers of cones survive despite their microenvironment and can provide a metabolic challenge to the diabetic retina.

Our cone measurements are likely to be dominated by M- and L-cones, since there are few S-cones near the fovea and a sparse distribution elsewhere. The decrease of cones found in the two younger diabetic subjects was greater than the numbers of S-cones at the locations measured. From previous visual function data with L- and M-cones in diabetic patients without extensive retinopathy, there is not a great loss of L- and M- cones, as evidenced by color matching in these subjects being accurate enough to provide quantitative parameters (5). However, changes to S-cones could alter cone distribution somewhat and visual function significantly, but results are mixed (4). Histology findings demonstrate structural changes to only S-cones, which clearly are not impacted by the yellowing of the human lens, yet may not be readily translated to testing with *in vivo* methods. The low numbers of S-cones in the fovea and sparse and irregular distribution elsewhere limit the potential impact of diabetes on foveal acuity, spatial resolution of white or long wavelength stimuli, or contrast sensitivity, as well as functions depending on numbers of cones. Similarly, the sparse distribution of S-cones would make difficult the comparison of small retinal regions, since there might not be S-cones in the sample. Functional tests that require S-cone input specifically and short wavelength light have been used. Conclusions for S-cone pathway damage providing a sensitive metric, or show more damage than occurs for M- or L-cone pathways, require individualized controls for removal of the short wavelength artifact due to pre-retinal filtering properties of the aging and diabetic lens.

Other early neuronal damage is described for the retina, which impacts on cone pathways. The cotton wool spots in diabetes have been shown to have longer term functional consequences, which likely have a negative impact on cone vision (10). Thinning of layers, as measured first with scanning laser polarimetry (9) and then OCT (11), has been shown in diabetic patients, particularly for inner retinal structures such as the retinal ganglion cells and the retinal nerve fiber layer. A carefully selected sample of Type 1 diabetics had a thickened retinal sublayer, the inner nuclear layer, early on in the course of the disease (37). The cone changes that we found occurred without thickness changes to total retinal thickness on OCT, which were reported on an individual patient basis rather than a group. A thickness metric has been shown to be related to cone changes (27, 29) in patients with sufficient pathological changes. Thus, the variability of a thickness metric may mask early vascular changes in diabetic eyes that might proceed or contribute to cone damage. Losses of either neural, glial, or vascular components were measurable without exudation leading to significant retinal thickening. More sensitive measures of retinal vascular changes, such as those provided by AOSLO imaging or OCTA, can help provide a better understanding of neuronal changes and neuronal-vascular coupling, and provide improved biomarkers for the management of diabetic eyes.

## Data Availability Statement

The raw data supporting the conclusions of this article will be made available by the authors, without undue reservation.

## Ethics Statement

The studies involving human participants were reviewed and approved by Indiana University Institutional Review Board. The patients/participants provided their written informed consent to participate in this study.

## Author Contributions

AE supervised the collection of the majority of the normative data, developed the model, guided the image processing tasks, sampled the diabetic data, graded images, analyzed the summary data, prepared statistics, wrote the manuscript, and made or designed the figures. BW collected the majority of the diabetic data on AOSLO and OCT, collected clinical data, analyzed OCT data on the diabetic subjects, maintained and queried the subject database for selection and quantification of subjects, processed AOSLO images, and edited the manuscript. RG processed images and made the AOSLO montages, selected cones montages for quantification, created a database for OCT and AOSLO data, and edited the manuscript. VP graded AOSLO montages, made figures, performed some of the statistics, and edited the manuscript. JP collected the OCT control data and provided summary statistics, assisted with plotting figures, and edited the manuscript. TG performed the examinations to qualify the diabetic patients, help select subjects for inclusion, helped design and performed OCT data collection and analysis, graded and interpreted images, and edited the manuscript. SB built the AOSLO apparatus, wrote the analysis software, helped select diabetic subjects, guided or performed the collection of diabetic and normative data, fine-tuned montaging, and edited the manuscript. All authors contributed to the article and approved the submitted version.

## Funding

Data collection and preparation of this article was supported by research grants from the National Eye Institute EY007624 to AE and EY024315 to SB.

## Conflict of Interest

The authors declare that the research was conducted in the absence of any commercial or financial relationships that could be construed as a potential conflict of interest.

## Publisher's Note

All claims expressed in this article are solely those of the authors and do not necessarily represent those of their affiliated organizations, or those of the publisher, the editors and the reviewers. Any product that may be evaluated in this article, or claim that may be made by its manufacturer, is not guaranteed or endorsed by the publisher.
